# Supplementary material for: Monospecific and bispecific monoclonal SARS-CoV-2 neutralizing antibodies that maintain potency against B.1.617
Source: Nat Commun. 2022 Mar 28;13:1638. doi: 10.1038/s41467-022-29288-3 (PMC8960874; doi:10.1038/s41467-022-29288-3)
Supplement: Supplementary file 1 — Supplementary Information [file 41467_2022_29288_MOESM1_ESM.pdf]

Supplementary Information

Peng et al.

Monospecific and bispecific monoclonal SARS-CoV-2 neutralizing antibodies that maintain potency against B.1.617

Supplementary Figures and Legends

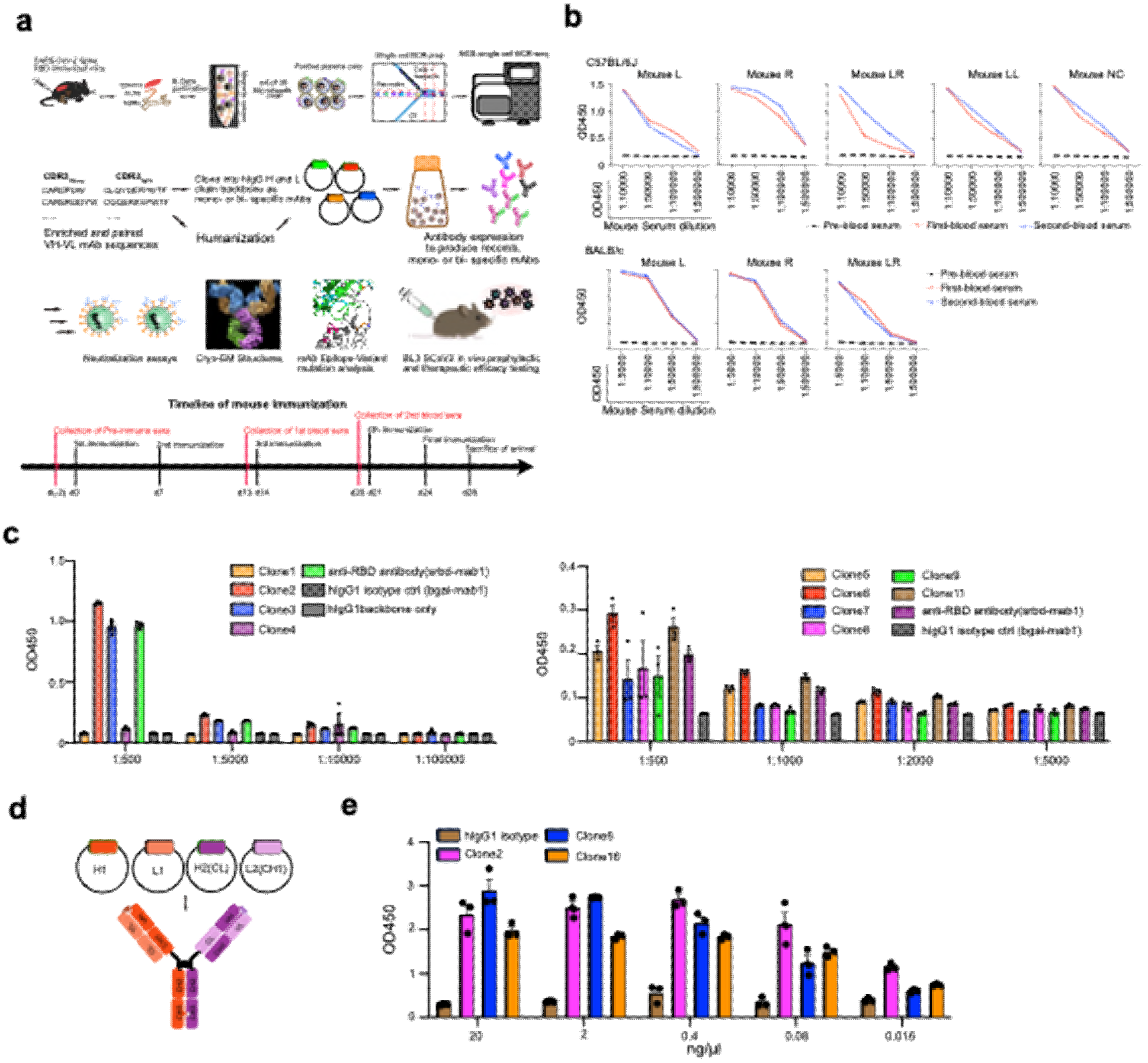

Supplementary Figure 1 | Measurement of mouse serum titers in SARS-CoV-2 Spike RBD immunized mice in this study.

a, Schematic of experiments. This schematic illustrates neutralizing antibody identification process through RBD-his tag protein mouse immunization-single B cell sequencing (**Top**), along with main assays of downstream analyses (**Middle**). The paired heavy chain and light chain sequences of the B cells were obtained using 10X Genomics VDJ sequencing. Antibodies were reconstructed by cloning of IgG heavy and light chains into human IgG1 backbone and expressed as recombinant monospecific or bispecific mAbs. Lead antibody clones were subjected to characterizations including neutralization assays, BL3 level anti- authentic SARS-CoV-2 efficacy testing, and structural analyses by Cryo-EM. (Bottom) A timeline of mouse immunization for antibody development.

b, SARS-CoV-2 RBD reactivity ELISA result of serum samples from different RBD immunized C57BL/6J (b) (n=5 mice) and BALB/c (c) mice (n=3 mice).

c, SARS-CoV-2 RBD reactivity ELISA result of recombinant monospecific mAb clones identified from single BCR sequencing of RBD immunized C57BL/6J (top) and BALB/c (bottom) mice. Data are presented as mean values +/- SEM, n=3 biological replicates.

d, Schematics of construct design and antibody structure of bispecific antibodies used in this study.

e, SARS-CoV-2 RBD reactivity ELISA result of top monospecific mAb clones (Clones 2 and 6) and a bispecific mAb clone (Clone 16). Data are presented as mean values +/- SEM, n=3 biological replicates.

Source data and additional statistics for experiments are provided in a supplemental excel file.

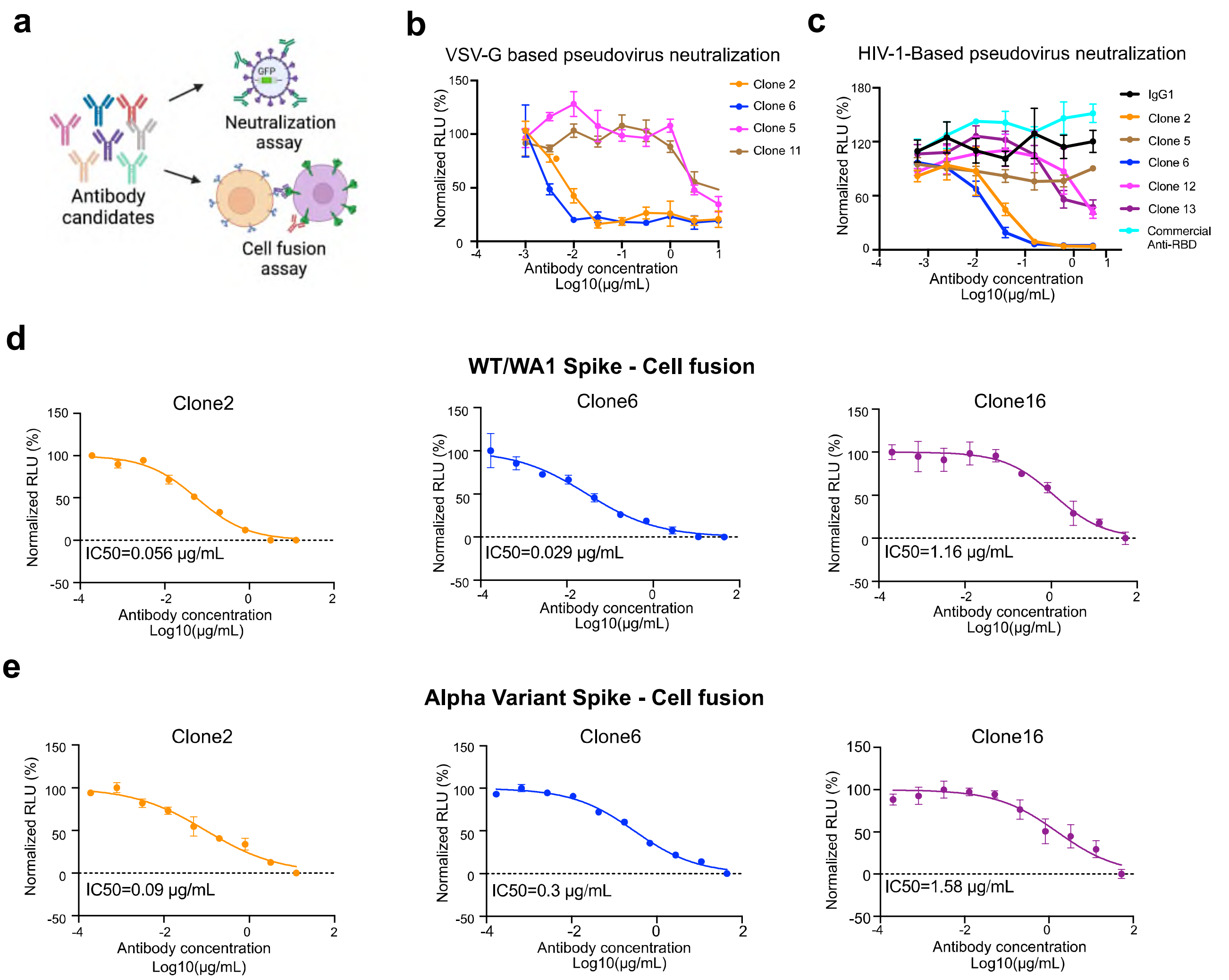

Supplementary Figure 2 | Neutralization capability testing of antibody clones with HIV1-based and VSV-G-based WT SARS-CoV-2 spike pseudotyped virus.

a, Schematics of initial mAb testing by neutralization assay and cell fusion assay.

b, Neutralization assay on Clone 2, Clone 6, Clone 5 and Clone 11 using WT SARS-CoV-2 Spike pseudotyped VSV- virus carrying a luciferase reporter, n=3 biological replicates.

c, Neutralization assay on Clone 2, Clone 5, Clone 6, Clone 12 and Clone 13, using WT SARS-CoV-2 Spike pseudotyped HIV-lentivirus carrying a luciferase reporter, n=3 biological replicates.

d, Cell fusion assay with SARS-CoV-2 Spike on top mAb clones, n=3 biological replicates.

e, Cell fusion assay with SARS-CoV-2 UK variant Spike on top mAb clones, n=3 biological replicates.

Source data and additional statistics for experiments are provided in a supplemental excel file.

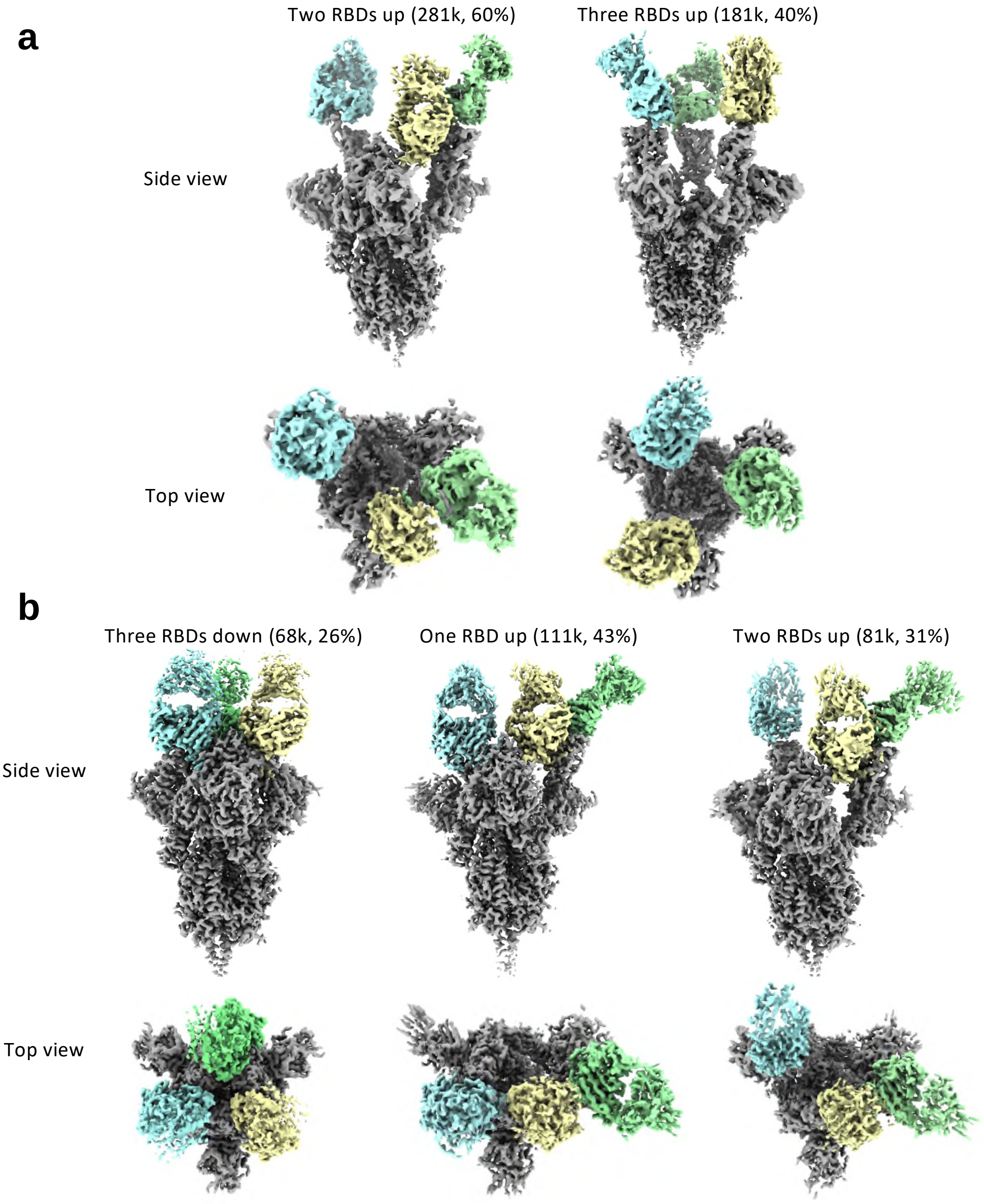

Supplementary Figure 3 | Cryo-EM density maps of the ectodomain of SARS-CoV-2 S trimer (gray) in complexes with Clone 2 (a) or Clone 6 (b) Fabs (green, yellow and cyan).

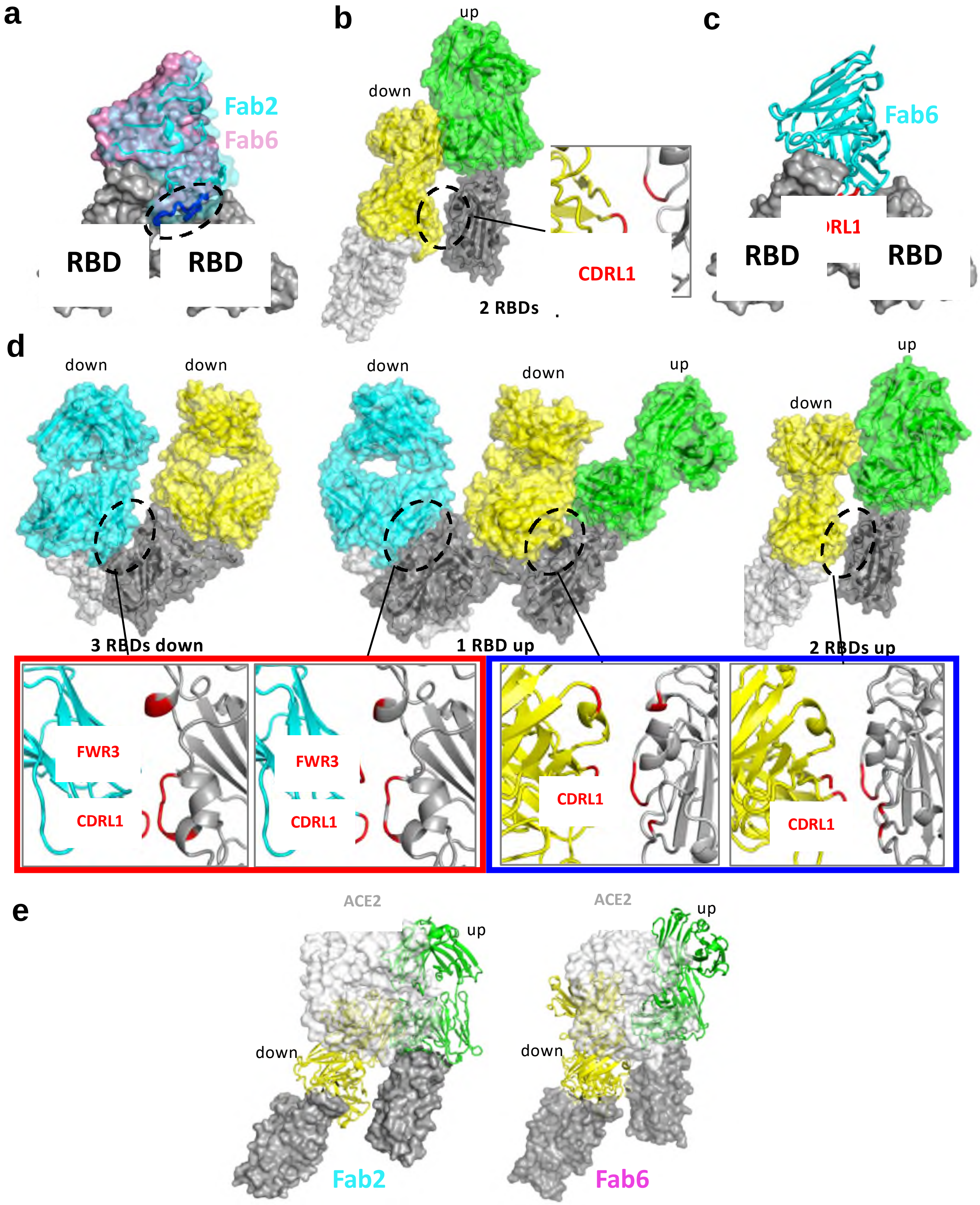

Supplementary Figure 4 | Additional binding interfaces between spike RBD and both clone Fabs.
a, Overlay of a down-RBD-bound Clone 2 Fab (cyan ribbons with transparent surface) onto a down-RBD-bound Clone 6 Fab (cyan surface) reveals a steric clash between the Clone 2 Fab and a neighboring RBD.

b, The additional binding interface between a down-RBD-binding Clone 2 Fab and an adjacent up-RBD in S trimers with 2 RBDs up.

c, The down-RBD-binding Clone 6 Fab sits on top of two adjacent down-RBDs in S trimers with 2 or 3 RBDs down. The CDRL1 loop highlighted in red inserts between two adjacent RBDs.

d, The additional binding interfaces between a down-RBD-binding Fab and adjacent down- or up-RBD in all Clone 6 Fab-S trimer complexes. The residues involved in the interactions are highlighted in red.

e, ACE2 bound to an up-RBD has additional steric clashes with a neighboring down-RBD bound Clone 2 (left) or Clone 6 (right) Fab in spike trimers with either 1 RBD up or 2 RBDs up. ACE2 is shown as light gray surface, Clone 2 and 6 Fabs are shown as yellow (on down-RBD) or green (on up-RBD) ribbons, and RBDs are shown as gray surfaces.

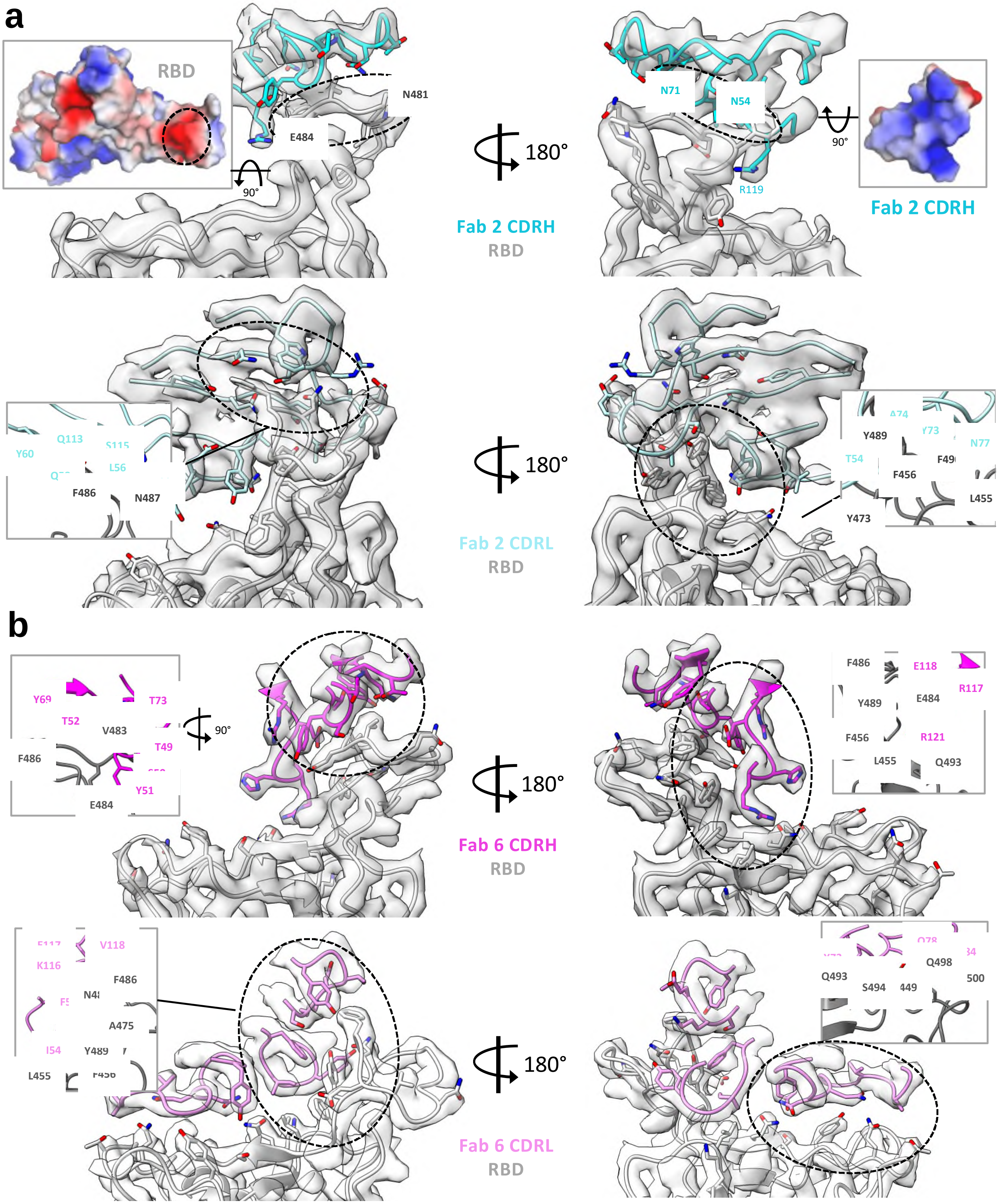

Supplementary Figure 5 | Detailed atomic interactions at the spike RBD-Fab interfaces.

a, Cryo-EM maps of the spike RBD binding interfaces with the Clone 2 Fab CDRH loops (upper panel) and CDRL loops (lower panel), with the fitted models in ribbon representation. The key interactions and

electrostatic surfaces (blue, positively charged; red, negatively charged) at the binding interfaces are shown in respective insets.

b, Cryo-EM maps of the spike RBD binding interfaces with the Clone 6 Fab CDRH loops (upper panel) and CDRL loops (lower panel), with the fitted models in ribbon representation. The key interactions are shown in respective insets.

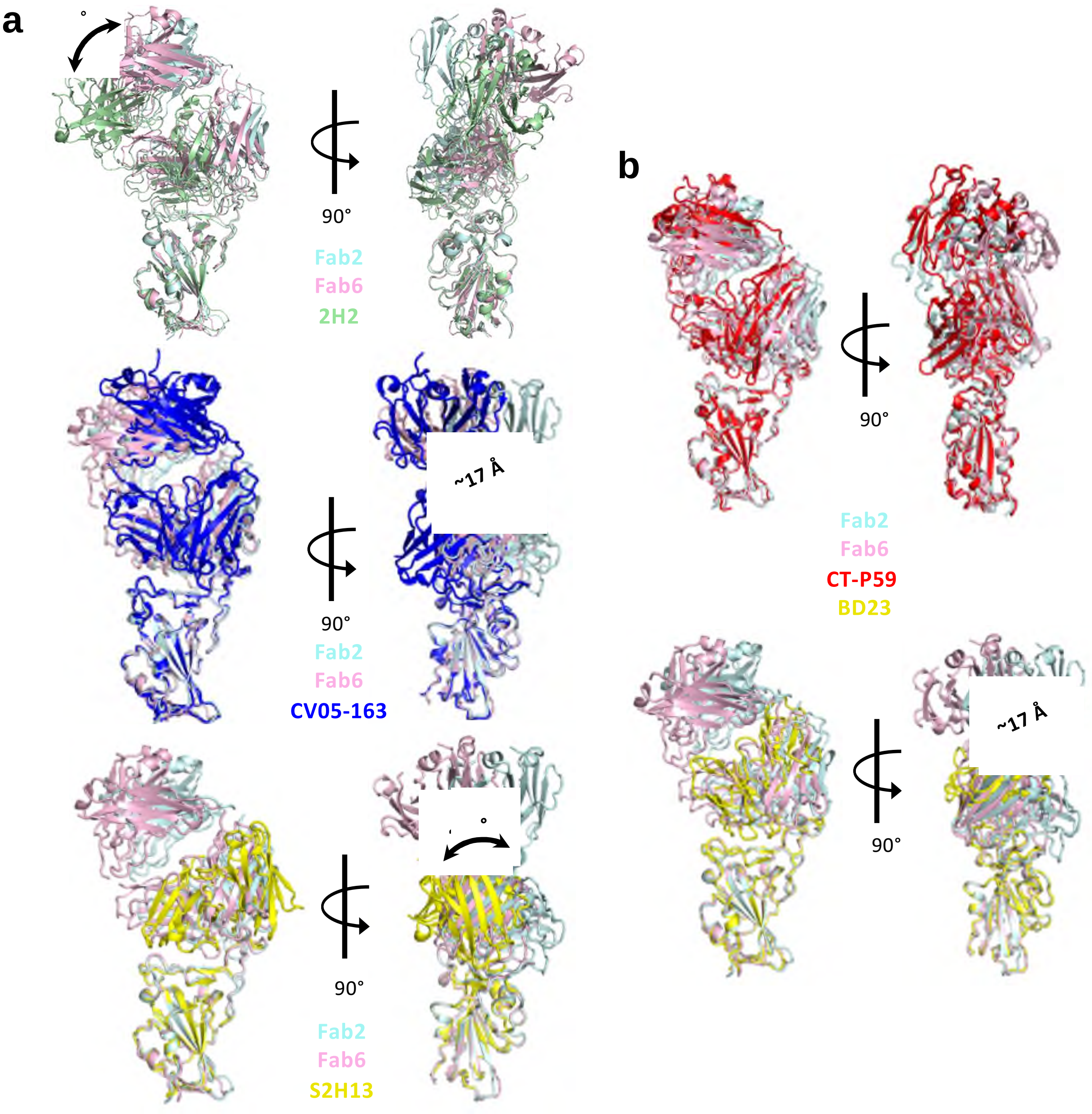

Supplementary Figure 6 | Comparison of binding orientations between Clone 2/6 and previous reported antibodies on SARS-CoV-2 spike RBD, with the RBD portions overlaid. a, Three previously reported antibodies bind the spike RBD in overall similar orientations as those of Clone 2/6, but with substantial rotations or shifts. **b**, Two previous reported antibodies resemble the binding conformations of Clone 2/6 on

spike RBD, however with the positions of heavy chains and light chains swapped. The PDB IDs of the published Fab-RBD structures: 2H2, 7DK5; CV05-163, 7LOP; S2H13, 7JV4; CT-P59, 7CM4; BD23, 7BYR.

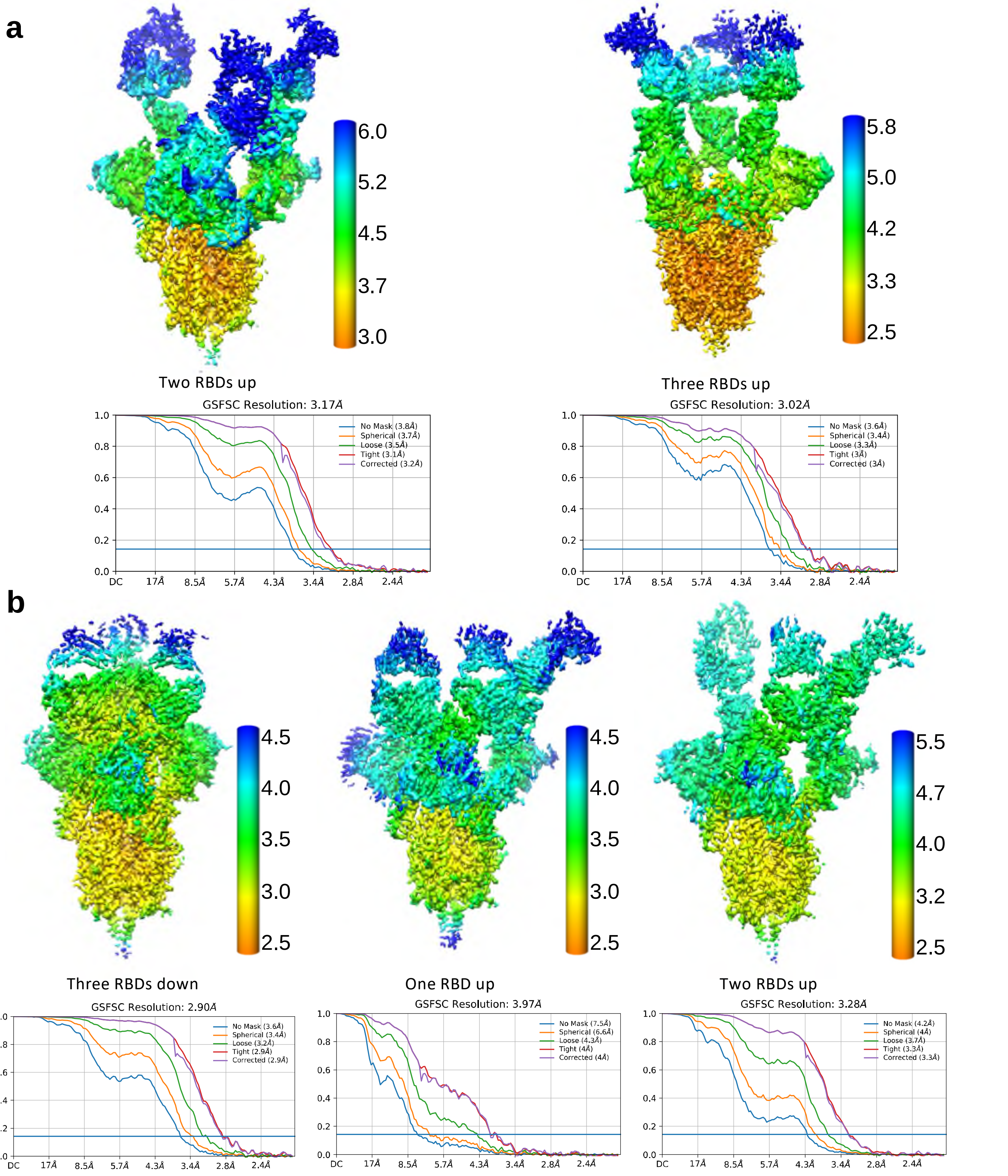

Supplementary Figure 7 | Local resolution estimations of the cryo-EM maps of SARS-CoV-2 S

ectodomain trimer (gray) in complexes with Clone 2 (a) or Clone 6 (b) Fabs. Fourier shell correlation (FSC) curves of the half-maps of each complex structure from gold standard refinement calculated by cryoSPARC are also shown, respectively.

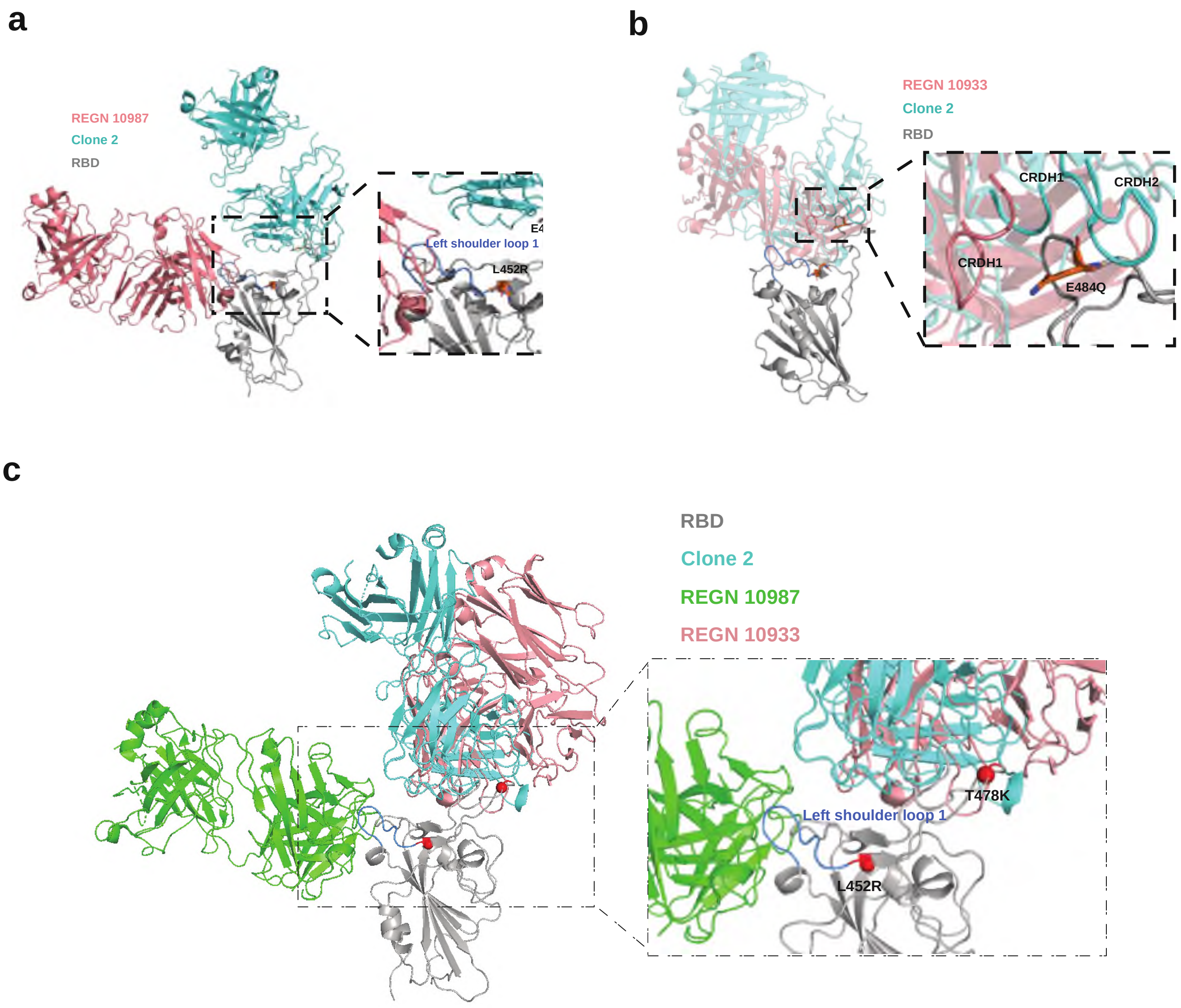

Supplementary Figure 8 | Epitope and mutation analysis of the Fab-spike RBD interfaces for lead mAb clone in comparison with representative existing clinical antibodies

Structural comparison of Clone 2, REGN 10933 and REGN 10987 revealed their distinct RBD epitopes and varied susceptibility to mutations of Delta variant. RBD structural model of Delta in complex with REGN 10987 (A, Pink, PDB: 6XDG), REGN 10933 (B, Pink, PDB: 6XDG) and Clone 2 (Cyan) is shown.

a, The epitope of REGN 10987 are mainly distributed in the left shoulder loop 1 (blue) region, which extends to L452R mutation site, while the clone 2 mainly targets the right ridge of RBD.

b, The paratopes of REGN 10933 and clones targeting the E484Q of RBD are labeled and shown. The remaining parts of the antibody structures were set 50% transparent.

c, Overlay of structures of REGN 10933, RGEN 10987 and Clone 2 Fab with RBD, with analysis of representative key residues.

Supplemental Table 1. List of oligos

Clone 1-VK-gBlcok	CACTAAGTCTTGCACTTGTCACGAATTCAATGGATATGCGGACACCCGCTCAATTTCTCGGGATTCTCCTCCTTTGGT<br>TTCCTGGCATCAAATGCGATATTAAGATGACACAAAGTCCTTCTTCTATGTACGCCAGCCTCGGCGAACGGGTCACC<br>ATAACTTGTAAGGCATCACAGGACATAAACTCATACCTTAGTTGGTTCCAACAGAAGCCAGGCAAAAGTCCTAAGA<br>CTCTGATATATCGCGCTAATCGATTGGTGGATGGGGTGCCCTCTCGCTTTTCCGGCAGTGGCTCCGGGCAAGACTAC<br>TCTCTCACAATCAGCAGCTTGGAGTATGAAGACATGGGAATATACTACTGCCTCCAGTATGATGAATTTCCTTGGAC<br>ATTTGGAGGCGGGACTAAATTGGAGATAAAGCGTACGGTGGCTGCACCATCTGTCTTCATC
Clone 2-VK-gBlcok	CACTAAGTCTTGCACTTGTCACGAATTCAATGGAATCAGATACACTCCTCCTCTGGGTGCTTCTTTTGTGGGTGCCTG<br>GGTCCACCGGGGACATTGTCCTTACCCAGTCACCCGCCTCCTTGGCAGTCAGTCTGGGACAAAGAGCCACTATAAGT<br>TGTCGGGCCTCTGAATCAGTTGAATACTACGGCACCTCTCTTATGCAATGGTATCAGCAAAAGCCCGGACAGCCTCC<br>TAAGGTACTCATTTACGCCGCAAGTAATGTAGAAAGTGGAGTGCCTGCCAGGTTCTCTGGCAGCGGAAGCGGTACA<br>GATTTCTCACTCAACATCCATCCTGTTGAAGAAGATGACATTGCCATGTATTTCTGCCAGCAAAGCAGGAAAGTGCC<br>TTGGACTTTCGGTGGTGGGACAAAGCTCGAAATAAAGCGTACGGTGGCTGCACCATCTGTCTTCATC
Clone 3-VK-gBlcok	CACTAAGTCTTGCACTTGTCACGAATTCAATGTCTGTCCCCACACAAGTTCTCGGCCTGCTGCTCCTTTGGCTCACAG<br>ATGCCCGGTGTGACATACAGATGACACAATCCCCCGCCAGTCTCTCAGCTTCCGTAGGGGAGACCGTAACCATTACC<br>TGCCGAGCATCCGAGAATATTTACTCAAACTTGGCATGGTATCAGCAGAAGCAAGGAAAAAGCCCCCAACTCCTGG<br>TTTACGTTGCCACAAATCTCGCCCACGGGGTCCCCTCAAGGTTCAGCGGTAGTGGCTCCGGGACCCAATACAGCCTG<br>AAAATAAATAGCCTTCAGTCTGAGGATTTCGGGTCCTACTATTGCCAACATTTCTGGGGGACCCCAACCTTTGGAAG<br>TGGGACCAAACTCGAAATCAAGCGTACGGTGGCTGCACCATCTGTCTTCATC
Clone 4-VK-gBlcok	CACTAAGTCTTGCACTTGTCACGAATTCAATGATGTCACCAGCTCAGTTCCTCTTCCTGTTGGTGCTCTGGATTCGAG<br>AAACTAACGGAGATGTCGTGATGACCCAAACCCCACTTACTCTCTCCGTGACTATCGGCCAACCCGCTAGTATATCA<br>TGTAAGTCATCTCAAAGTTTGTTGGATTCCGACGGCAAGACATATCTTAATTGGCTGCTGCAACGCCCAGGACAGTC<br>CCCTAAGAGACTGATCTATCTCGTGAGTAAGCTCGATAGCGGGGTCCCTGACCGATTCACTGGTAGTGGAAGCGGTA<br>CTGATTTTACCCTGAAGATTTCTCGGGTCGAGGCTGAGGACCTCGGTGTATATTATTGTTGGCAGGGAACTCACTTCC<br>CATACACCTTCGGAGGCGGTACAAAGCTGGAAATAAAACGTACGGTGGCTGCACCATCTGTC
Clone 5-VK-gBlcok	CACTAAGTCTTGCACTTGTCACGAATTCAATGGTGTTCACCCCCCACATCCTGGGCCTGCTGCTGTTCTGGATCAGCG<br>CCAGCACCGGCGACATCCTGCTGACCCAGAGCCCCGCCACCCTGAGCGTGACCCCCGGCGAGACCGTGAGCCTGAG<br>CTGCAGGGCCAGCCAGAGCATCTACAAGAACCTGCACTGGTACCAGCAGAAGAGCCACAGGAGCCCCAGGCTGCTG<br>ATCAAGTACGCCAGCGACAGCATCAGCGGCATCCCCAGCAGGTTCACCGGCAGCGGCAGCGGCACCGACTACACCC<br>TGAGCATCAACAGCGTGAAGCCCGAGGACGAGGGCATCTACTACTGCCTGCAGGGCTACAGCACCCCCCTGACCTT<br>CGGCGCCGGCACCAAGCTGGAGCTGAAGAGGACGGTGGCTGCACCATCTGTCTTCATC
Clone 6-VK-gBlcok	CACTAAGTCTTGCACTTGTCACGAATTCAATGGAGAAAGATACCCTCTTGCTCTGGGTACTGCTGCTCTGGGTGCCTG<br>GGTCCACCGGCGACATTGTGCTGACACAATCACCTGCCTCCCTGGCAGTAAGTCTGGGCCAACGGGCAACCATCTCT<br>TGCCGGGCTAGCGAAAGTGTAGATAATTACGGGATCTCTTTCATGAATTGGTTTCAACAAACACCTGGGCAGCCTCC<br>CAAACTGCTCATCTACGGCTCAAGTAACCAAGGTAGTGGTGTGCCTGCTAGGTTTTCAGGTTCCGGGAGTGGCACAG<br>ACTTCAGTCTGAACATACACCCAATGGAAGAAGACGATACTGCTATGTATTTCTGTCAACAGAGTAAGGAGGTACC<br>ATATACATTCGGAGGCGGAACCAAACTCGAAATCAAGAGGACGGTGGCTGCACCATCTGTCTTCATC
Clone 7-VK-gBlcok	CACTAAGTCTTGCACTTGTCACGAATTCAATGATGAGCCCCGCCCAGTTCCTGTTCCTGCTGGTGCTGTGGATCAGGG<br>AGACCAACGGCGACGTGGTGATGACCCAGACCCCCCTGACCCTGAGCGTGACCATCGGCCAGCCCGCCAGCATCAG<br>CTGCAAGAGCAGCCAGAGCCTGCTGGACAGCGACGGCAAGACCTACCTGAACTGGCTGCTGCAGAGGCCCGGCCAG<br>AGCCCCAAGAGGCTGATCTACCTGGTGAGCAAGCTGGACAGCGGCGTGCCCGACAGGTTCACCGGCAGCGGCAGCG<br>GCACCGACTTCACCCTGAAGATCAGCAGGGTGGAGGCCGAGGACCTGGGCGTGTACTACTGCTGGCAGGGCACCCA<br>CTTCCCCTACACCTTCGGCGGCGGCACCAAGCTGGAGATCAAGAGGACGGTGGCTGCACCATCTGTCTTCATC

Clone 8-VK-gBlcok

CACTAAGTCTTGCACTTGTCACGAATTCAATGAAGCTGCCCGTGAGGCTGCTGGTGCTGATGTTCTGGATCCCCGCC
AGCAGCAGCGACGTGCTGATGACCCAGACCCCCCTGAGCCTGCCCGTGAGCCTGGGCGACCAGGCCAGCATCAGCT
GCAGGAGCAGCCAGAGCATCGTGCACAGCAACGGCAACACCTACCTGGACTGGTACCTGCAGAAGCCCGGCCAGA
GCCCCAAGCTGCTGATCTACAAGGTGAGCAACAGGTTCAGCGGCGTGCCCGACAGGTTCAGCGGCAGCGGCAGCGG
CACCGACTTCACCCTGAAGATCAGCAGGGTGGAGGCCGAGGACCTGGGCGTGTACTACTGCTTCCAGGGCAGCCAC
GTGCCCTGGACCTTCGGCGGCGGCACCAAGCTGGAGATCAAGAGGACGGTGGCTGCACCATCTGTCTTCATC

Clone 9-VK-gBlcok

CACTAAGTCTTGCACTTGTCACGAATTCAATGATGAGCAGCGCCCAGTTCCTGGGCCTGCTGCTGCTGTGCTTCCAG
GGCACCAGGTGCGACCTGCAGATGACCCAGACCACCAGCAGCCTGAGCGCCAGCCTGGGCGACAGGGTGACCATCA
ACTGCAGGGCCAGCCAGGACATCACCAACTACCTGAACTGGTACCAGCAGAAGCCCGACGGCACCCTGAAGCTGCT
GATCTACTACACCAGCAGGCTGCCCAGCGGCGTGCCCAGCAGGTTCAGCGGCAGCGGCAGCGGCACCGACTACAGC
CTGACCATCAGCAACCTGGAGCAGGAGGACATCGCCACCTACTTCTGCCAGCAGGGCAAGACCTTCCCCTGGACCTT
CGGCGGCGGCACCAAGCTGGAGATCAAGAGGACGGTGGCTGCACCATCTGTCTTCATC

Clone 10-VK-gBlcok

CACTAAGTCTTGCACTTGTCACGAATTCAATGGAGAGCCAGATCCAGGCCTTCGTGTTCGTGTTCCTGTGGCTGAGC
GGCGTGGACGGCGACATCGTGATGACCCAGAGCCACAAGTTCATGAGCACCAGCGTGGGCGACAGGGTGAGCATCA
CCTGCAAGGCCAGCCAGGACGTGAGCAGCGCCGTGGCCTGGTACCAGCAGAAGCCCGGCCAGAGCCCCAAGGTGCT
GATCTACTGGGCCAGCACCAGGCACACCGGCGTGCCCGACAGGTTCACCGGCAGCGGCAGCGGCACCGACTACACC
CTGACCATCAGGAGCGTGCAGGCCGAGGACCTGGCCCTGTACTACTGCCAGCAGCACTACAACACCCCCAGGACCT
TCGGCGGCGGCACCAAGCTGGAGATCAAGAGGACGGTGGCTGCACCATCTGTCTTCATC

Clone 11-VK-gBlcok

CACTAAGTCTTGCACTTGTCACGAATTCAATGCGCCCATCTATACAGTTTTTGGGACTTCTTCTGTTCTGGCTTCATG
GAGCACAATGCGATATACAAATGACCCAGTCCCCCTCCTCCCTCAGCGCCAGTTTGGGCGGCAAAGTCACTGTTACT
TGCAAGGCCTCTCAGGACATAAACAAGTATATCGCTTGGTATCAACACAAGCCCGGTAAGGGACCACGGCTTCTGA
TTCACTATACATCAACACTTCAACCAGGGATTCCCTCAAGATTCTCCGGCAGCGGTTCAGGGAGGGATTACTCATTC
AGTATCAACAATCTGGAACCTGAGGATATAGCCACATACTACTGCTTGCAATACGACAATTTGTACACATTCGGAGG
TGGGACTAAGCTGGAGATGAAACGCACGGTGGCTGCACCATCTGTCTTCATC

Clone 12-VK-gBlcok

CACTAAGTCTTGCACTTGTCACGAATTCAATGGAATCTGATACATTGCTTCTTTGGGTGCTCTTGCTGTGGGTTCCAG
GCTCCACTGGAGATATTGTTATGACCCAAAGCCCTGATAGCCTCGCCGTCTCCCTGGGCGAGCGGGCGACGATTAAC
TGCAGGGCATCAGAGAGTGTTGAATACTATGGTACAAGTCTCATGCAGTGGTATCAGCAAAAGCCCGGTCAGCCTC
CGAAATTGTTGATATACGCAGCGTCCAACGTGGAATCCGGGGTGCCATCACGGTTCTCCGGTTCCGGTAGTGGTACG
GACTTCACCTTCACCATTTCCTCTCTGCAGCCCGAGGATATTGCCACATATTACTGTCAACAAAGTCGGAAAGTTCCA
TGGACCTTCGGGGGTGGGACTAAAGTGGAGATAAAGCGAACGGTGGCTGCACCATCTGTCTTCATC

Clone 13-VK-gBlcok

CACTAAGTCTTGCACTTGTCACGAATTCAATGGAGTCCGATACCCTTCTCCTGTGGGTGCTGTTGCTTTGGGTCCCAG
GCAGTACGGGAGATATTCAGATGACACAATCCCCCTCTAGCCTGTCAGCGTCCGTGGGCGACCGAGTTACGATTACC
TGCCGAGCATCAGAATCCGTAGAGTATTATGGCACGTCTCTTATGCAATGGTATCAGCAGAAACCGGGTAAAGCGC
CAAAACTGCTCATCTACGCGGCGTCTAACGTGGAGTCAGGTGTACCCTCCAGATTTTCTGGTTCAGGAAGCGGGACT
GATTTTACATTGACTATAAGCTCACTGCAACCGGAAGATTTCGCTACCTACTATTGTCAACAATCACGGAAGGTCCC
ATGGACCTTCGGGCAGGGTACTAAAGTCGAGATTAAAAGGACGGTGGCTGCACCATCTGTCTTCATC

UK varaint gBlock3	CCAGGGCTGGCTGTCTGATTGGAGCAGAGCATGTGAACAACTCCTATGAGTGTGACATCCCAATTGGAGCAGGCAT<br>CTGTGCCTCCTACCAGACCCAGACCAACAGCCATAGGAGGGCAAGGTCTGTGGCAAGCCAGAGCATCATTGCCTAC<br>ACAATGAGTCTGGGAGCAGAGAACTCTGTGGCTTACAGCAACAACAGCATTGCCATCCCAATTAACTTCACCATCTC<br>TGTGACCACAGAGATTCTGCCTGTGAGTATGACCAAGACCTCTGTGGACTGTACAATGTATATCTGTGGAGACAGCA<br>CAGAGTGTAGCAACCTGCTGCTCCAATATGGCTCCTTCTGTACCCAACTTAACAGGGCTCTGACAGGCATTGCTGTG<br>GAACAGGACAAGAACACCCAGGAGGTGTTTGCCCAGGTGAAGCAGATTTACAAGACACCTCCAATCAAGGACTTTG<br>GAGGCTTCAACTTCAGCCAGATTCTGCCTGACCCAAGCAAGCCAAGCAAGAGGTCCTTCATTGAGGACCTGCTGTTC<br>AACAAGGTGACCCTGGCTGATGCTGGCTTCATCAAGCAATATGGAGACTGTCTGGGAGACATTGCTGCCAGGGACC<br>TGATTTGTGCCCAGAAGTTCAATGGACTGACAGTGCTGCCTCCACTGCTGACAGATGAGATGATTGCCCAATACACC<br>TCTGCCCTGCTGGCTGGCACCATCACCTCTGGCTGGACCTTTGGAGCAGGAGCAGCCCTCCAAATCCCATTTGCTAT<br>GCAGATGGCTTACAGGTTCAATGGCATTGGAGTGACCCAGAATGTGCTCTATGAGAACCAGAAACTGATTGCCAAC<br>CAGTTCAACTCTGCCATTGGCAAGATTCAGGACTCCCTGTCCAGCACAGCCTCTGCCCTGGGCAAACTCCAAGATGT<br>GGTGAACCAGAATGCCCAGGCTCTGAACACCCTGGTGAAGCAAC
South African-gBlock1	cactatagggagacccaagctggctagccaccATGTTTGTGTTCCTGGTGCTGCTGCCACTGGTGTCCAGCCAGTGTGTGAACTTCACC<br>ACCAGGACCCAACTTCCTCCTGCCTACACCAACTCCTTCACCAGGGGAGTCTACTACCCTGACAAGGTGTTCAGGTC<br>CTCTGTGCTGCACAGCACCCAGGACCTGTTCCTGCCATTCTTCAGCAATGTGACCTGGTTCCATGCCATCCATGTGTC<br>TGGCACCAATGGCACCAAGAGGTTTGCCAACCCTGTGCTGCCATTCAATGATGGAGTCTACTTTGCCAGCACAGAGA<br>AGAGCAACATCATCAGGGGCTGGATTTTTGGCACCACCCTGGACAGCAAGACCCAGTCCCTGCTGATTGTGAACAAT<br>GCCACCAATGTGGTGATTAAGGTGTGTGAGTTCCAGTTCTGTAATGACCCATTCCTGGGAGTCTACTACCACAAGAA<br>CAACAAGTCCTGGATGGAGTCTGAGTTCAGGGTCTACTCCTCTGCCAACAACTGTACCTTTGAATATGTGAGCCAAC<br>CATTCCTGATGGACTTGGAGGGCAAGCAGGGCAACTTCAAGAACCTGAGGGAGTTTGTGTTCAAGAACATTGATGG<br>CTACTTCAAGATTTACAGCAAACACACACCAATCAACCTGGTGAGGGGCCTGCCACAGGGCTTCTCTGCCTTGGAAC<br>CACTGGTGGACCTGCCAATTGGCATCAACATCACCAGGTTCCAGACCCTGCTGGCTCTGCACATTTCCTACCTGACA<br>CCTGGAGACTCCTCCTCTGGCTGGACAGCAGGAGCAGCAGCCTACTATGTGGGCTACCTCCAACCAAGGACCTTCCT<br>GCTGAAATACAATGAGAATGGCACCATCACAGATGCTGTGGACTGTGCCCTGGACCCACTGTCTGAGACCAAGTGT<br>ACCCTGAAATCCTTCACAGTGGAGAAGGGCATCTACCAGACCAGCAACTTCAGGGTCC
South African-gBlock2	CCAGCAACTTCAGGGTCCAACCAACAGAGAGCATTGTGAGGTTTCCAAACATCACCAACCTGTGTCCATTTGGAGAG<br>GTGTTCAATGCCACCAGGTTTGCCTCTGTCTATGCCTGGAACAGGAAGAGGATTAGCAACTGTGTGGCTGACTACTC<br>TGTGCTCTACAACTCTGCCTCCTTCAGCACCTTCAAGTGTTATGGAGTGAGCCCAACCAAACTGAATGACCTGTGTTT<br>CACCAATGTCTATGCTGACTCCTTTGTGATTAGGGGAGATGAGGTGAGACAGATTGCCCCTGGACAAACAGGCAAC<br>ATTGCTGACTACAACTACAAACTGCCTGATGACTTCACAGGCTGTGTGATTGCCTGGAACAGCAACAACCTGGACAG<br>CAAGGTGGGAGGCAACTACAACTACCTCTACAGACTGTTCAGGAAGAGCAACCTGAAACCATTTGAGAGGGACATC<br>AGCACAGAGATTTACCAGGCTGGCAGCACACCATGTAATGGAGTGAAGGGCTTCAACTGTTACTTTCCACTCCAATC<br>CTATGGCTTCCAACCAACCTACGGAGTGGGCTACCAACCATACAGGGTGGTGGTGCTGTCCTTTGAACTGCTCCATG<br>CCCCTGCCACAGTGTGTGGACCAAAGAAGAGCACCAACCTGGTGAAGAACAAGTGTGTGAACTTCAACTTCAATGG<br>ACTGACAGGCACAGGAGTGCTGACAGAGAGCAACAAGAAGTTCCTGCCATTCCAACAGTTTGGCAGGGACATTGCT<br>GACACCACAGATGCTGTGAGGGACCCACAGACACTGGAGATTCTGGACATCACACCATGTTCCTTTGGAGGAGTGT<br>CTGTGATTACACCTGGCACCAACACCAGCAACCAGGTGGCTGTGCTCTACCAGGATGTGAACTGTACTGAGGTGCCT<br>GTGGCTATCCATGCTGACCAACTTACACCAACCTGGAGGGTCTACAGCACAGGCAGCAATGTGTTCCAGACCAGGG<br>CTGGCTGTCTGATTGGAGCAGAGCATGTGAACAACTCCTATGAGTGTGACATCCCAATTGGAGCAGGCATCTGTGCC<br>TCCTACCAGACCCAGACCAACAGCCCAAGGAGGGCAAGGTCTGTGGCAAGCCAGAGCATCATTGCCTACACAATGA<br>GTCTGGGAGTGGAGAACTCTGTGGCTTACAGCAACAACAGCATTGCCATCCCAACCAACTTCACCATCTCTGTGACC<br>ACAGAGATTCTGCCTGTGAGTATGACCAAGACCTCTGTGGACTGTACAATGTATATCTGTGGAGACAGCACAG
del19R1	cttaagcttggtaccgagctcggatccTCAACAACAGGAGCCACAGGAACAAC
del19F1	GCTAGACTGGACAAGGTGGAGGC

AGAATGCCCAGGCTCTGAACACCCTGGTGAAGCAACTTTCCAGCAACTTTGGAGCCATCTCCTCTGTGCTGAATGACATCCTGAGCAGACTGGA
CAAGGTGGAGGCTGAGGTCCAGATTGACAGACTGATTACAGGCAGACTCCAATCCCTCCAAACCTATGTGACCCAACAACTTATCAGGGCTGCT
GAGATTAGGGCATCTGCCAACCTGGCTGCCACCAAGATGAGTGAGTGTGTGCTGGGACAAAGCAAGAGGGTGGACTTCTGTGGCAAGGGCTA
CCACCTGATGAGTTTTCCACAGTCTGCCCCTCATGGAGTGGTGTTCCTGCATGTGACCTATGTGCCTGCCCATGAGAAGAACTTCACCACAGCCC
CTGCCATCTGCCATGATGGCAAGGCTCACTTTCCAAGGGAGGGAGTGTTTGTGAGCAATGGCACCCACTGGTTTGTGACCCAGAGGAACTTCTA
TGAACCACAGATTATCACCACAGACAACACCTTTGTGTCTGGCAACTGTGATGTGGTGATTGGCATTGTGAACAACACAGTCTATGACCCACTCC
AACCTGAACTGGACTCCTTCAAGGAGGAACTGGACAAATACTTCAAGAACCACACCAGCCCTGATGTGGACCTGGGAGACATCTCTGGCATCA
ATGCCTCTGTGGTGAACATCCAGAAGGAGATTGACAGACTGAATGAGGTGGCTAAGAACCTGAATGAGTCCCTGATTGACCTCCAAGAACTGG
GCAAATATGAACAATACATCAAGTGGCCATGGTACATCTGGCTGGGCTTCATTGCTGGACTGATTGCCATTGTGATGGTGACCATAATGCTGTG
TTGTATGACCTCCTGTTGTTCCTGTCTGAAAGGCTGTTGTTCCTGTGGCTCCTGTTGTAAGTTTGATGAGGATGACTCTGAACCTGTGCTGAAAG
pVP28gB4 GAGTGAAACTGCACTACACCTGAggatccgagctcggtaccaagcttaag

CCAACCTGGAGGGTCTACAGCACAGGCAGCAATGTGTTCCAGACCAGGGCTGGCTGTCTGATTGGAGCAGAGCATGTGAACAACTCCTATGAG
TGTGACATCCCAATTGGAGCAGGCATCTGTGCCTCCTACCAGACCCAGACCAACAGCAGGAGGAGGGCAAGGTCTGTGGCAAGCCAGAGCAT
CATTGCCTACACAATGAGTCTGGGAGCAGAGAACTCTGTGGCTTACAGCAACAACAGCATTGCCATCCCAACCAACTTCACCATCTCTGTGACCA
CAGAGATTCTGCCTGTGAGTATGACCAAGACCTCTGTGGACTGTACAATGTATATCTGTGGAGACAGCACAGAGTGTAGCAACCTGCTGCTCCA
ATATGGCTCCTTCTGTACCCAACTTAACAGGGCTCTGACAGGCATTGCTGTGGAACAGGACAAGAACACCCAGGAGGTGTTTGCCCAGGTGAA
GCAGATTTACAAGACACCTCCAATCAAGGACTTTGGAGGCTTCAACTTCAGCCAGATTCTGCCTGACCCAAGCAAGCCAAGCAAGAGGTCCTTC
ATTGAGGACCTGCTGTTCAACAAGGTGACCCTGGCTGATGCTGGCTTCATCAAGCAATATGGAGACTGTCTGGGAGACATTGCTGCCAGGGAC
CTGATTTGTGCCCAGAAGTTCAATGGACTGACAGTGCTGCCTCCACTGCTGACAGATGAGATGATTGCCCAATACACCTCTGCCCTGCTGGCTG
GCACCATCACCTCTGGCTGGACCTTTGGAGCAGGAGCAGCCCTCCAAATCCCATTTGCTATGCAGATGGCTTACAGGTTCAATGGCATTGGAGT
GACCCAGAATGTGCTCTATGAGAACCAGAAACTGATTGCCAACCAGTTCAACTCTGCCATTGGCAAGATTCAGGACTCCCTGTCCAGCACAGCC
pVP28gB3 TCTGCCCTGGGCAAACTCCAAGATGTGGTGAACCAGAATGCCCAGGCTCTGAACA

GGCATCTACCAGACCAGCAACTTCAGGGTCCAACCAACAGAGAGCATTGTGAGGTTTCCAAACATCACCAACCTGTGTCCATTTGGAGAGGTGT
TCAATGCCACCAGGTTTGCCTCTGTCTATGCCTGGAACAGGAAGAGGATTAGCAACTGTGTGGCTGACTACTCTGTGCTCTACAACTCTGCCTCC
TTCAGCACCTTCAAGTGTTATGGAGTGAGCCCAACCAAACTGAATGACCTGTGTTTCACCAATGTCTATGCTGACTCCTTTGTGATTAGGGGAGA
TGAGGTGAGACAGATTGCCCCTGGACAAACAGGCAAGATTGCTGACTACAACTACAAACTGCCTGATGACTTCACAGGCTGTGTGATTGCCTG
GAACAGCAACAACCTGGACAGCAAGGTGGGAGGCAACTACAACTACAGGTACAGACTGTTCAGGAAGAGCAACCTGAAACCATTTGAGAGGG
ACATCAGCACAGAGATTTACCAGGCTGGCAGCACACCATGTAATGGAGTGCAGGGCTTCAACTGTTACTTTCCACTCCAATCCTATGGCTTCCAA
CCAACCAATGGAGTGGGCTACCAACCATACAGGGTGGTGGTGCTGTCCTTTGAACTGCTCCATGCCCCTGCCACAGTGTGTGGACCAAAGAAG
AGCACCAACCTGGTGAAGAACAAGTGTGTGAACTTCAACTTCAATGGACTGACAGGCACAGGAGTGCTGACAGAGAGCAACAAGAAGTTCCT
GCCATTCCAACAGTTTGGCAGGGACATTGCTGACACCACAGATGCTGTGAGGGACCCACAGACCTTGGAGATTCTGGACATCACACCATGTTCC
TTTGGAGGAGTGTCTGTGATTACACCTGGCACCAACACCAGCAACCAGGTGGCTGTGCTCTACCAGGGAGTGAACTGTACTGAGGTGCCTGTG
pVP28gB2 GCTATCCATGCTGACCAACTTACACCAACCTGGAGGGTCTACAGC

cactatagggagacccaagctggctagccaccATGTTTGTGTTCCTGGTGCTGCTGCCACTGGTGTCCAGCCAGTGTGTGAACCTGACCACCAGGACCCAA
CTTCCTCCTGCCTACACCAACTCCTTCACCAGGGGAGTCTACTACCCTGACAAGGTGTTCAGGTCCTCTGTGCTGCACAGCACCCAGGACCTGTT
CCTGCCATTCTTCAGCAATGTGACCTGGTTCCATGCCATCCATGTGTCTGGCACCAATGGCACCAAGAGGTTTGACAACCCTGTGCTGCCATTCA
ATGATGGAGTCTACTTTGCCAGCACAGAGAAGAGCAACATCATCAGGGGCTGGATTTTTGGCACCACCCTGGACAGCAAGACCCAGTCCCTGC
TGATTGTGAACAATGCCACCAATGTGGTGATTAAGGTGTGTGAGTTCCAGTTCTGTAATGACCCATTCCTGGACGTCTACTACCACAAGAACAA
CAAGTCCTGGATGAAGTCTGAGTTCAGGGTCTACTCCTCTGCCAACAACTGTACCTTTGAATATGTGAGCCAACCATTCCTGATGGACTTGGAG
GGCAAGCAGGGCAACTTCAAGAACCTGAGGGAGTTTGTGTTCAAGAACATTGATGGCTACTTCAAGATTTACAGCAAACACACACCAATCAAC
CTGGTGAGGGACCTGCCACAGGGCTTCTCTGCCTTGGAACCACTGGTGGACCTGCCAATTGGCATCAACATCACCAGGTTCCAGACCCTGCTGG
CTCTGCACAGGTCCTACCTGACACCTGGAGACTCCTCCTCTGGCTGGACAGCAGGAGCAGCAGCCTACTATGTGGGCTACCTCCAACCAAGGAC
CTTCCTGCTGAAATACAATGAGAATGGCACCATCACAGATGCTGTGGACTGTGCCCTGGACCCACTGTCTGAGACCAAGTGTACCCTGAAATCC
pVP28gB1 TTCACAGTGGAGAAGGGCATCTACCAGACCAGCAACTTC

1
