# Supplementary material for: Monospecific and bispecific monoclonal SARS-CoV-2 neutralizing antibodies that maintain potency against B.1.617
Source: Nat Commun. 2022 Mar 28;13:1638. doi: 10.1038/s41467-022-29288-3 (PMC8960874; doi:10.1038/s41467-022-29288-3)
Supplement: Supplementary file 3 — Description Supplementary Data [file 41467_2022_29288_MOESM3_ESM.docx]

**Supplementary Dataset 1**

This dataset provides the single cell BCR sequencing processed data of the sequences and clonotypes of immunized mice.
